# Time to recovery from Eclampsia and its determinants in east Gojjam zone hospitals, Amhara, Ethiopia, 2017/18

**DOI:** 10.1186/s12884-021-03769-7

**Published:** 2021-04-14

**Authors:** Bekalu Kassie, Yibelu Bazezew, Yewbmirt Sharew, Leltework Yismaw, Melaku Desta, Muluneh Alene

**Affiliations:** 1 Department of Midwifery, College of Health Sciences, Debre Markos University, Debre Markos, Ethiopia; 2 Department of Public Health, College of Health Sciences, Debre Markos University, Debre Markos, Ethiopia

**Keywords:** Eclampsia, Pregnancy induced hypertension, Time to recovery, East Gojjam, Ethiopia

## Abstract

**Background:**

Eclampsia is a tonic clonic type of seizure among pre-eclamptic mothers. Time to recovery from eclampsia is to mean that the time when the mother recovered from severity features of pre-eclampsia. As far as the mother is not free from severity features, she is in a potential to end-up with repeated seizure (eclampsia). Therefore, combating eclampsia through controlling severity features is crucial to enhance maternal health quality, reduce maternal morbidity and mortality, and improve prenatal outcomes. There was no literature that describes the recovery time of eclampsia and its determinants in Ethiopia. Therefore, this study aimed to assess the recovery time from eclampsia and its determinants in East Gojjam zone hospitals.

**Methods:**

An institutional based retrospective follow up study was conducted between January 2014 and December 2017 among 608 eclamptic mothers in East Gojjam zone Hospitals. Simple random sampling technique was used. Data were coded and entered to Epidata version 3.1 and was exported to SPSS version 20 and then to Stata 14. We used the adjusted hazard ratio (AHR) with 95% confidence interval at *p*-value less than 0.05 to measure strength of association.

**Result:**

The median recovery time of eclampsia was 12 h with inter-quartile range of (1–48 h). The rate of recovery from eclampsia among mothers aged more than 20 years was reduced by half (AHR 0.50 (0.28, 0.89)) than the teenagers. The rate of recovery from eclampsia among mothers who had prolonged labor was 1.3 times (AHR 1.26 (1.01, 1.57)) than those whose labor was less than 12 h. About 32% of mothers with multiple convulsions recoverd later than (AHR 0.68 (0.52, 0.87)) those who had single convulsion. As compared to antepartum convulsion, the rate of recovery from postpartum eclampsia was 1.8 times faster (AHR 1.81(1.17, 2.81)).

**Conclusion:**

The median recovery time from severity features among eclamptic mothers in East Gojjam zone hospitals was half a day. It is affected by age, duration of labor, number of convulsions and time of occurrence of the event. Special attention for elders, prevent recurrent convulsion and faster termination for the antepartum eclamptic mothers are recommended from this follow-up study.

## Introduction

Improving maternal and child health is a global priority [1] .The problem of maternal health during pregnancy is one with many special features as maternity is not a disease. Pregnancy and childbirth are privileged functions of women, essential for the survival of our species which is not being compared with other burdens of disease [2]. Maternal mortality is a key indicator of international development, and its reduction has long been a challenge in low-income countries, despite the existence of effective interventions [3].

Pregnancy and childbirth related complications are common problems that cause many women to die and suffer life-threatening difficulties that disable them [4–6]. Even if most of maternal deaths are preventable, maternal mortality rate is still high and it is the major public health problem in the world, especially in developing countries [7–9]. Globaly in the past two and a half decades, there was a significant progress in the declining maternal mortality [4, 5, 7]. In spite of this improvement, more than 289,000 women still die each year as a result of pregnancy and childbirth through pregnancy to the first 42 days of the postpartum period globally [4, 5, 7, 9]. Nearly all of these women live in poor nations where 99% of the global maternal death has been occurring in developing countries, mainly in the two developing regions, sub-Saharan Africa and southern Asia [5, 7]. Eclampsia is the development of generalized grandmal tonic clonic convulsions in a pregnant or puerperal woman, usually between 20 weeks’ of gestation and the first 48 h of postpartum period, mostly in a woman with gestational hypertension or preeclampsia in the absence of other neurologic conditions [10, 11]. It is the most devastating type of Hypertension Disorders of pregnancy (HDP) [10].

Hypertensive disorders during pregnancy remains a significant public health problem throughout the world, and eclampsia is one of the most life-threatening complications of these disorders. HDP accounts for nearly 18% of all maternal deaths worldwide, with an estimated 62,000–77,000 deaths per year (18). Eclampsia is a major health problem in developing countries and every year, eclampsia is associated with an estimated 50,000 maternal deaths Worldwide [12].

Around 70% of mothers with eclamsia develop life threatening complications which include abruption placentae, disseminated intravascular coagulopathy, acute renal failure, hepatocellular injury, liver rupture, intracerebral hemorrhage, transient blindness, cardio respiratory arrest, aspiration pneumonitis, acute pulmonary edema, postpartum hemorrhage and HELLP syndrome (Hemolysis, Elevated Liver enzymes, Low Platelet counts).

Ethiopia, is one of the six countries which has highest Maternal Mortality Ratios (MMR) in the world and currently, the Ethiopian MMR is 420/100,000 live births [13]. Most causes of these deaths are preventable including eclampsia. In Ethiopia, there is an overall increasing trend of maternal death due to hypertensive disorder of pregnancy [14–17].

Maternal Mortality Ratios (MMR) in Amhara Region are calculated at 266 deaths per 100,000 live births. Of the total maternal deaths recorded in the region, 8.1% is as the result of hypertensive disorder of pregnancy, which is the third common cause of maternal mortality following hemorrhage and anemia of pregnancy [17].

Delay in health care-seeking, distance to facility, delay in initiation of treatment, severity of complications, absence of antenatal care attendance and quality of care contributes to the majority of HDP related maternal deaths [18].

Reduction of maternal mortality is a priority program in the international arena and ending preventable maternal mortality is the main concern agenda of the sustainable developmental goals. Sustainable Developmental Goal (SDG), Goal 3, target 3.1 declared that MMR would be reduced to less than 70/100,000 live births by 2030, with no single country having an MMR greater than 140 [9, 19].

One of the activities is prevention, early detection and treatment of pregnancy related complications (9). To reduce maternal mortality rates, the government of Ethiopia is working by assigning trained health professional to all districts, giving training and refreshment course, allocating ambulance service to all districts and developing well organized referral linkage in all regions. Despite all these efforts, MMR is one of the highest in the world and Eclampsia still remains one of the leading causes of direct maternal mortality, even if it is preventable. So doing a research in this area is invaluable to provide evidence based practice to enhance maternal health, reduce maternal morbidity and mortality, and improve prenatal outcomes and also to improve the care in health care tier system because the outcome of Eclampsia is time dependent.

As of the authors’ effort, there is no literature that describes the recovery time of eclampsia and its determinants in Ethiopia but also in the Africa. Therefore, this study was aimed to assess the average recovery time of eclampsia and its determinants in East Gojjam zone hospitals of Amhara region among women admitted with a diagnosis of eclampsia. Furthermore, the findings of this study will be helpful for health providers, police makers and other stakeholders and will be very helpful to be used as a baseline for further researches and also for educational purposes.

## Methods

All methods were carried out in accordance with relevant guidelines and regulations from study design to the analysis.

### Study design and setting area

We conducted an institutional based retrospective follow up study to determine time to recovery of eclampsia and its determinants between January 2014 to December 2017 in East Gojjam zone hospitals. This zone is bordered on the south by the Oromia Region, on the west by Weast Gojjam, on the north of south Gondar, and on the east by South Wollo and the bend of the Abay River defines the zone’s northern, eastern and southern boundaries. Its highest point is mount Choke. Based on the 2007 census conducted by the central statistical agency of Ethiopia, this zone has a total population of 2,153,937. In the administrative zone there are around 111 health institutions of which 8 are primary and 1 is referral hospitals. This study was conducted infour hospitals by taking mothers with eclampsia from 3 primary hospitals (Shegaw Motta Hospital, Bichena primary Hospital and Lumame primary hospital) and Debre Markos referal hospital.

In hospitals where we conducted this study, similar standard of treatment of eclamptic mother was there. All eclamptic women managed with antihypertensive and anticonvulsant medications besides the general and emergency managements. The commonly used anticonvulsant was magnesium sulphate while the antihypertensive was hydralazine. The target of management for these women is controlling severity features and keep vital organs well functioning.

**Study population:** All pregnant and postpartum mothers who were admitted to hospitals of the East Gojjam zone with the diagnosis of eclampsia within the last five years.

### Eligibility criteria

**Inclusion Criteria:** mothers admitted to selected hospitals with a diagnosis of eclampsia during the study period were included.

### Sample size determination

The sample size was determined by using sample size determination formula for time to event data based on the Cox’s proportional hazards model sample size calculation with the assumptions of the null hypothesis (_Ho_): b = 0 and Alternative hypothesis (H_a_): b ≠ 0.

1 $$ n=\frac{{\left({Z}_{\alpha /2}+{Z}_{\beta}\right)}^2}{b^2{p}_1{p}_2d} $$

n = the required sample size; *Z*_*α*/2_ = critical value of the standard and normal distributed variable at 5% significance level; *Z*_*β*_ = critical value of standard normal distributed variable at 20% of *β*; *β* = the probability of type two error, b = log (hazard ratio), *p*_1_ = the proportion of the number of patients in the first category, *p*_2_ = the proportion of number of patients in the second category and d represents the proportion of the event. In this case the proportion of recovered patients from eclampsia, d = 97.1%, which has been taken from a previous study [18].

The sample size was calculated by using the above formula considers six key factors based on a study conducted in Hawassa [18] and finally the maximum sample size is taken which is 552 and by adding 10% for those incomplete patient charts the final sample size become 608.

### Sampling procedure and technique

To select the required sample size, simple random sampling technique used. Data collectors select all eclamptic cases from a list of the patients’ registration books between January 2014 to December 2017. Then samples allocated proportionally to each selected hospital. From all admitted eclamptic mothers in the selected hospitals (Debre Markos comprehensive specialized referral hospital, lumame primary hospital, Bichena primary hospital and Shegaw Mota General hospital) six hundred eight samples were selected proportionally.

### Operational definition

**Recovery**: mothers diagnosed with eclampsia and has no cerebral symptoms AND creatinine level less than 1.1 mg/dl AND blood pressure less than 150/100 mmHg, AND Aspartet aminotransferase and alanine aminotransferase enzymes level not more than two fold of normal range. AND is to indicate if the mother has either of the features the conclusion should be not recovered.

**Time to recovery (survival time/ time-to-event):** is the time from diagnosis of eclampsia to recovery.

**Starting time:** Time of diagnosis.

**End time:** Time of discharge of the patient/last recored on the patient chart/.

**Censored**: Lost to follow up and referred cases.

### Data collection tools and procedures

Data were collected from the patient’s chart by preparing a checklist which contains five major groups of variables: Maternal socio-demographic characteristics, laboratory results, management approaches, clinical symptoms/signs, and maternal and fetal outcomes until discharge. Eight midwives collected the data; two for each hospital after 2-days training given.

### Data quality control

To assure the data quality, data collection instruments designed with high emphasis. Regular meetings held between data collectors, supervisor and the principal investigator to discuss and solve problematic issues. We reviewed and checked the data for completeness before data entry.

### Data processing and analysis

The collected data were coded and entered to Epidata version 3.1 and was exported to SPSS version 20 and then to STATA 14 for data clearance, transformation (recoding variables) and analysis purpose. In this study, the outcome/variable of interest was time until the mother recovered from severty features of pre-eclampsia. Hence, survival analysis was used to estimate survivor and/or hazard functions from survival data, to compare survivor and/or hazard functions and to assess the relationship between the explanatory variables to survival time. We used Kaplan-Meier to estimate the distribution of recovery time and to observe the experience of recovery time among each group for categorical covariates. Log-rank test was used for comparing the survival (recovery) experience between two or more groups. To identify determinant factors for recovery time, semi-parametric (Cox proportional hazard) and parametric methods used. Factors significantly associated with the outcome variable in single covariate analysis at *p*-value less than 0.2 were included in the multiple covariate analysis and statistically significant associations were measured by the Adjusted hazard ratio (AHR) with 95% confidence interval at *p*-value less than 0.05.

## Result

In this retrospective follow-up study, about charts of 586 mothers admitted in 4 selected hospitals with the diagnosis of eclampsia between January 2014 to December 2017 were involved with a response rate of 96.4%. Twenty-two of the reviewed charts were with incomplete information and considered as non-response rate. The median age of the study participants was 27 years and about 32% of them were in the age group of 25–29 years. More than 58% of them were rural residents. Among all eclamptic mothers who visited selected hospitals in East Gojjam zone, 47.8% were primiparous. Around 37 % of participants faced eclampsia at a gestational age of 33 weeks or earlier. Of the total participants, 6.8% gave birth to twins while the rest were singletons (Table 1).

**Table 1:** Socio-demographic and reproductive factors description among eclamptic mothers in East Gojjam zone hospitals

Variables	Categories	Frequency	Percent
Age in years	16–19	21	3.6
	20–24	151	25.8
	25–29	191	32.6
	30–34	107	18.3
	35–39	90	15.4
	> = 40	26	4.4
Residence	Urban	244	41.6
	Rural	342	58.4
Parity	Nulliparous	12	2.1
	Primiparous	280	47.8
	Multiparous	218	37.2
	Grand multiparous	76	13.0
Gestational age	<=33 wks	215	36.7
	34–36 weeks	140	23.9
	> = 37 weeks	231	39.4
Type of pregnancy	Singleton	546	93.2
	Twin	40	6.83
ANC follow-up	Yes	525	89.6
	No	61	10.4
Mode of delivery	SVD	377	64.3
	Assisted delivery	209	35.7
Place of delivery	Home	30	5.12
	Institution	556	94.9
Duration of labor (464)	<=12 h	310	66.8
	> 12 h	154	33.2
Initiation of labor	Induced	288	50.0
	Spontaneous	288	50.0
Number of convulsions	Single	443	75.6
	Multiple	143	24.4
Convulsion to administration of anticonvulsant	Less than an hour	428	73.0
	More than an hour	158	27.0

### Clinical management approaches

All eclamptic mothers are managed based on the national guidline which includes mainly three components. First component is the general management which includes emergency mamnagement, progress assessment, and documentation. From the progress assessment, neurological evaluation score at admission by Glasgow Coma Scale (GCS) was the one. The minimum score of GSC was 2 with 0.5% and the maximum was 15 with mean score of 13.03.

For controlling convulsion all mothers given anticonvulsant medications. About 94.4% of eclampsic mothers managed with magnesium sulphate while the rest took diazepam as anticonvulsant medication. For controlling hypertension, 80.50% of women given anti hypertensive while the 19.5% didn’t take any antihypertensive medication. Of those who took antihypertensive, majority (68,4%) were given hydralyzin followed by methyledopa (65.2%) and nefidipine (12.3%). From the total 586 eclamptic mothers, 25.8% gave birth by cesarean section for the indications of uncontrolled eclampsia (40.4%), failed inductionnon (29.1%), non-reassuring fetal heart rate (25.5%), fetomarernal disproportion (6.6%) and antepartum hemorrhage (3.97%).

The definitive management of eclampsia was termination of pregnmancy. For termination cesarean section is not the only option rather induction of labor is also considerd as one mechanism of termination. All the decisions for termination options are based on the indications and pre-requesites. Majority (55.63%) of women undergone with cesarean section took general anesthesia. From the total eclamptic mothers 79.4% had live births while the rest 20.6% lost their fetuses. Not only losing their fetuses but also they lost their lives (3.9%).

In reduction of maternal mortality magnesium sulphate is better than diazepam. Of the total 553 mother who took magnesium sulphate only 20 (3.6%) died while from 38 participants who took diazepam 15.79% (*n* = 6) died.

Age category, gestational age, duration of labor, number of convulsions and convulsion to administration of anticonvulsant were significantly different in survival time at 5% level of significance.

**Fig. 1:**
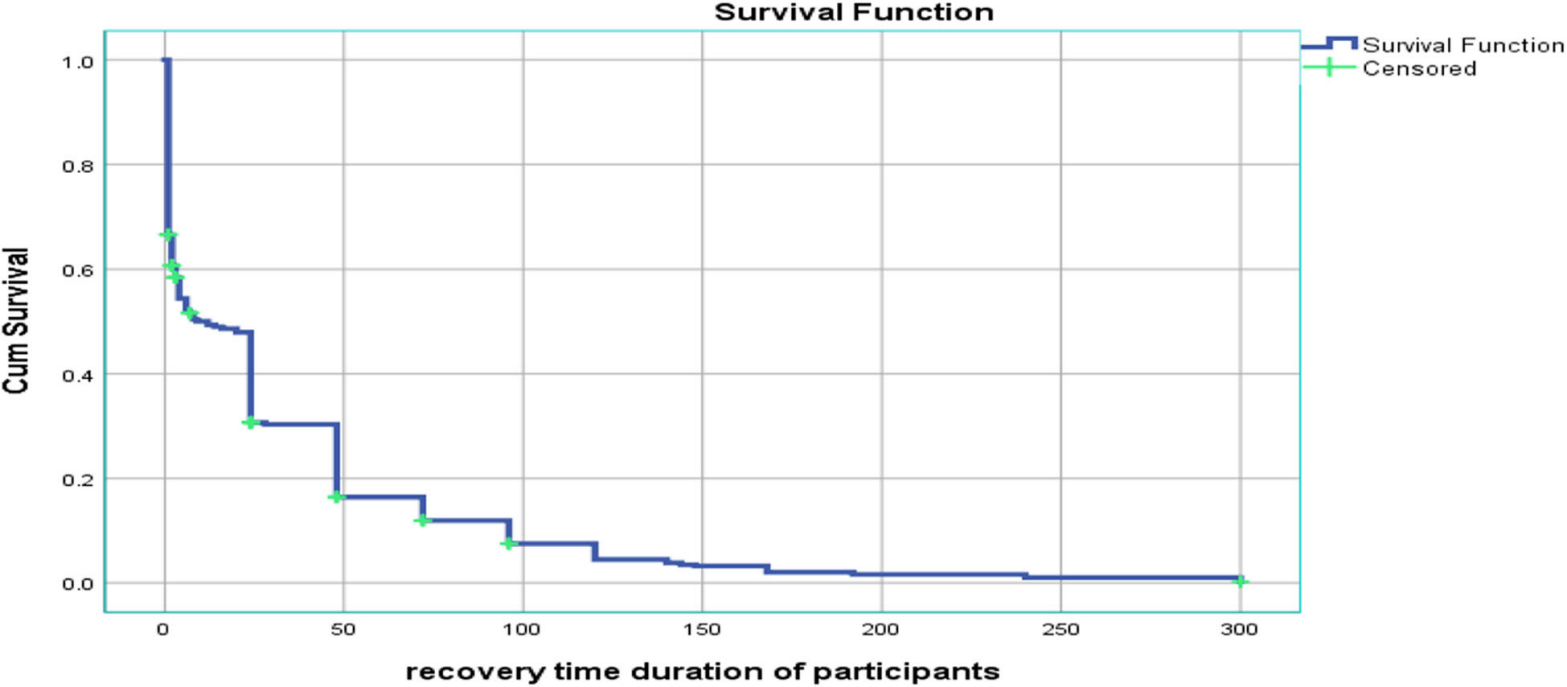
Survival function graph of the recovery time from eclampsia among eclamptic mothers in east Gojjam zone hospitals, 2018

Median recovery time of mothers from eclampsia varied among various categories of variables. For instance, in age, it varies from an hour to 1 day (Table 2). The survival curve tends to decrease rapidly within half a day indicating that most mothers have recovered from the problem within 12 h and slower over time (Fig. 1). Relatively it is in line with a study in Finland [20].

**Table 2:** Comparison of survival time by using log rank test among eclamptic mothers in East Gojjam zone hospitals, 2018

Variables	Categories	Frequency	Median recovery time from eclampsia (hours)	Log rank test	
				X2	*P*-value
Age in years	16–19	21 (3.6)	1	12.05	0.03
	20–24	151 (25.8)	14		
	25–29	191 (32.6)	24		
	30–34	107 (18.3)	4		
	35–39	90 (15.4)	4		
	> = 40	26 (4.4)	8		
Residence	Urban	244 (41.6)	8	0.77	0.38
	Rural	342 (58.4)	12		
Parity	Nulliparous	12 (2.1)	24	0.96	0.81
	Primiparous	280 (47.8)	14		
	Multiparous	218 (37.2)	8		
	Grand multiparous	76 (13.0)	4		
Gestational age	<=33 weeks	215 (36.7)	24	6.98	0.03
	34–36 weeks	140 (23.9)	4		
	> = 37 weeks	231 (39.4)	6		
Type of pregnancy	Singleton	546 (93.2)	12	0.39	0.53
	Twin	40 (6.83)	4		
ANC follow-up	Yes	525 (89.6)	7	0.69	0.40
	No	61 (10.4)	24		
Mode of delivery	SVD	377 (64.3)	20	0.83	0.66
	C/S	148 (25.3)	4		
	Assisted	61 (10.4)	24		
Place of delivery	Home	30 (5.12)	3	0.01	0.93
	Institution	556 (94.9)	12		
Duration of labor (464)	<=12 h	310 (66.8)	24	7.45	0.01
	> 12 h	154 (33.2)	3		
Initiation of labor	Induced	288 (50.0)	14	1.45	0.23
	Spontaneous	288 (50.0)	8		
Number of convulsions	Single	443 (75.6)	4	13.79	0.001
	Multiple	143 (24.4)	24		
Convulsion to administration of anticonvulsant	Less than an hour	428 (73.0)	6	4.01	0.045
	More than an hour	158 (27.0)	24

In the multivariable analysis, age of participants, duration of labor, number of convulsions and time of occurrence of convulsion found to be significantly associated with the time of recovery from eclampsia. The hazard of recovering late from eclampsia was about 50% among those aged from 20 to 24 years as compared to teenagers. Similarly, mothers aged from 25 to 29 years were 0.48 times to recover slowly from eclampsia as compared to those aged from 16 to 19.

Mothers who were in labor for more than 12 h were 1.26 times more likely to recover faster from eclampsia than those who were in labor for less than 12 h. Number of convulsion was the one factor that can increase the hazard of delaying the recovery from eclampsia. Mothers with multiple convulsion were 0.68 times more likely to recover too late from this problem.

**Fig. 2:**
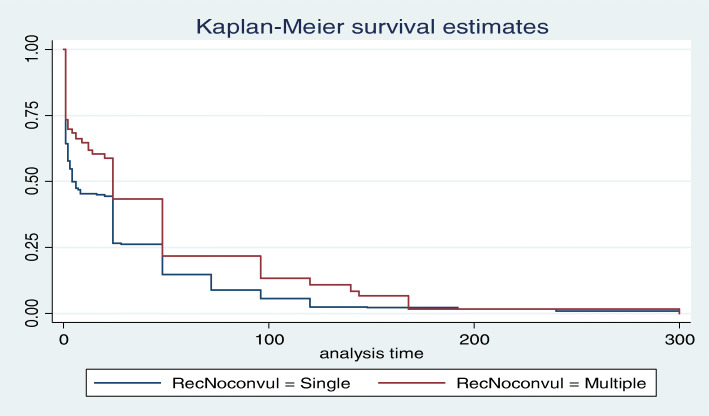
Kaplan-Meier Survival estimate of time to recovery among single and multiple convulsion groups 2018

The rate of recovery from eclampsia among mothers aged more than 20 years was reduced by half AHR 0.50 (0.28, 0.89) than the teenagers at any time in the follow up period. Those mothers who had prolonged labor was 1.3 times AHR 1.26 (1.01, 1.57) higher than those whose labor was less than 12 h to recover from the disease. The rate of recovery from Eclampsia among mothers with multiple convulsions were 32% AHR 0.68 (0.52, 0.87) reduced than those who had single convulsion (Fig. 2). The rate of recovery of mothers from eclampsia among those who developed the disease at the postpartum period was 1.8 times AHR 1.81(1.17, 2.81) higher than the antepartum eclampsia (Table 3).

**Table 3:** Predictors of time to recovery from eclampsia in East Gojjam zone hospitals, 2018: a cox regression report

Variable	Category	CHR (95% CI)	AHR (95% CI)	*P*-value
Age in years	16–19	1	1	
	20–24	0.55 (0.35, 0.88)	**0.50 (0.28, 0.89)**	**0.021**
	25–29	0.62 (0.39, 0.97)	**0.48 (0.26, 0.87)**	**0.016**
	30–34	0.65 (0.41, 1.03)	0.53 (0.28, 1.01)	0.053
	35–39	0.55 (0.34, 0.88)	**0.40 (0.21, 0.77)**	**0.006**
	> = 40	0.68 (0.38, 1.21)	**0.45 (0.20, 0.99)**	**0.049**
Residence	Urban	1	1	
	Rural	0.94 (0.79, 1.11)	0.96 (0.77, 1.19)	0.722
Parity	Nulliparous	1		
	Primiparous	1.16 (0.65, 2.07)		
	Multiparous	1.21 (0.68, 2.18		
	Grand multiparous	1.12 (0.61, 2.07)		
Gestational age	<=33 weeks	1	1	
	34–36 weeks	1.08 (0.87, 1.30		
	> = 37 weeks	1.24 (1.02, 1.50)		
Type of pregnancy	Singleton	1		
	Twin	1.09 (0.78, 1.52)		
ANC follow-up	Yes	1		
	No	0.91 (0.69, 1.19)		
Mode of delivery	SVD	1		
	C/S	1.06 (0.88, 1.29)		
	Assisted delivery	096 (0.72, 1.27)		
Place of delivery	Home	1		
	Institution	1.01 (0.69, 1.48)		
Duration of labor (464)	<=12 h	1	1	
	> 12 h	1.25 (1.02, 1.52)	**1.26 (1.01, 1.57)**	**0.036**
Initiation of labor	Induced	1		
	Spontaneous	1.09 (0.92, 1.29)		
Number of convulsions from	Single	1		
	Multiple	0.73 (0.60, 0.89)	**0.68 (0.52, 0.87)**	**0.003**
Convulsion to administration of anticonvulsant	Less than an hour	1		
	More than an hour	0.85 (0.71, 1.03)		
Time of occurrence of convulsion	Antepartum	1	1	
	Intrapartum	1.54 (1.18, 2.01)	1.35 (0.97, 1.88)	0.076
	Postpartum	1.22 (0.91 1.63)	**1.81 (1.17, 2.81)**	**0.008**

## Discussion

The overall median recovery time from eclampsia (first documentation of the diagnosis to recovery from severity features) in this study was half a day. The rate of recovery from eclampsia among mothers aged more than 20 years was reduced by half as compared to teenagers at any time in the follow up period. This might be because the younger the age, the more organs to cope the disease. Meaning, organs of these young people have good capacity to cope the disorder than elders because survival of a patient depends on the damage level of vital organs.

Mothers who were in labor for more than 12 h were 1.26 times more likely to recover faster from eclampsia than those who were in labor for less than 12 h. In other words, mothers in labor for more than 12 h were 26% more to recover from eclampsia than those who stayed for less than 12 h in labor.

Number of convulsion was one of the factors that can increase the hazard of delaying the recovery from eclampsia. Mothers with multiple convulsion were 0.68 times more to recover from this problem, which means 32% of mothers with multiple convulsions recovered later than mothers with singleconvulsion (Fig. 2). This might be because of the reason that the higher the number convulsions, the higher risk of vital organ damage which easily imply low rate of recovery.

Those mothers who had prolonged labor was 1.3 times higher than those whose labor was less than 12 h to recover from the disease. This might be because women whose convulsion controlled are expected to give birth vaginaly by induction that takes more time to deliver while non-controlled ones are expected to give birth through cesarean section. So if not controlled there will be delayed recovery.

The rate of recovery of mothers who developed eclampsia during the postpartum period was 1.8 times higher than those who developed antepartum eclampsia. Since termination of pregnancy is the definitive management of eclampsia, postpartum disease is less likely to proceed because the cause already terminated.

In general overall average time of recovery from eclampsia after admission to the health institutions in East Gojjam zone was short. This is affected by recored time since we cannot get the actual start time of the convulsion. Age dependant management, Prevent recurrent convulsion and special attention for antepartum convulsion are recommended. Prospective forllowup could strentgthen the output.

### Limitation of the study

Since the study was a retrospective follow-up in nature, missing variables were there and many variables are out of analysis because of incompleteness. The start time of the follow-up was time of diagnosis of the patient as eclampsia and documented and therefore, this may reduce the duration because of either late diagnosis or late documentation.

Since authors cannot get similar study findings, discussion in comparison with other studies not yet done.

## Data Availability

The datasets used and/or analyzed during the current study are available from the corresponding author on reasonable request.

## Abbreviations

AHR: Adjusted hazard ratio
HDP: Hypertensive disorders of pregnancy
